# *Diplopterys pubipetala* (Malpighiaceae): Insights into Antioxidant, Antibacterial, and Antifungal Activities with Chemical Composition Analysis via UHPLC-MS/MS and GC/MS

**DOI:** 10.3390/molecules30040946

**Published:** 2025-02-18

**Authors:** Veronica de Melo Sacramento, Vanessa de Andrade Royo, Pedro Henrique Fonseca Veloso, Kamila Soares Freitas Souto, Alisson Samuel Portes Caldeira, Carlos Henrique Gomes Martins, Sara Lemes de Souza, Ezequias Pessoa de Siqueira, Fernando Ribeiro Cassiano, Afrânio Farias de Melo Júnior, Dario Alves de Oliveira, Elytania Veiga Mnezes, Tânia Maria de Almeida Alves

**Affiliations:** 1 Natural Products Laboratory, State University of Montes Claros, Montes Claros 39401-089, MG, Brazil; veronica.sacramento.2014@gmail.com (V.d.M.S.); pedrofonsecambc@gmail.com (P.H.F.V.); kamilasoares08@gmail.com (K.S.F.S.); 2 Laboratory of Bioactive Natural Products Chemistry, René Rachou Institute, Oswaldo Cruz Foundation, Belo Horizonte 30190-002, MG, Brazil; alisson.caldeira@fiocruz.br (A.S.P.C.); ezequias.siqueira@fiocruz.br (E.P.d.S.); tania.alves@fiocruz.br (T.M.d.A.A.); 3 Antimicrobial Testing Laboratory, Institute of Biomedical Sciences, Federal University of Uberlândia, Uberlândia 38405-320, MG, Brazil; carlos.martins2@ufu.br (C.H.G.M.); lea.demic@icbim.ufu.br (S.L.d.S.); 4 Department of Exact Sciences, Center for Exact and Technological Sciences, State University of Montes Claros, Montes Claros 39401-089, MG, Brazil; fernando.cassiano@unimontes.br; 5 Bioprospecting and Genetic Resources Laboratory, State University of Montes Claros, Montes Claros 39401-089, MG, Brazil; afraniofariasdemelo@gmail.com (A.F.d.M.J.); dario.aol@gmail.com (D.A.d.O.); menezes.elytania@gmail.com (E.V.M.)

**Keywords:** biological properties, natural products, bioactive compounds, antimicrobial activity, therapeutic potential

## Abstract

*Diplopterys pubipetala* (Malpighiaceae) is a liana native to the Brazilian Cerrado biome, traditionally used in Ayahuasca preparations. Despite its cultural importance, research on its chemical composition and biological activities, which may have therapeutic potential, is limited. This study investigated the volatile and non-volatile secondary metabolites of *D. pubipetala* leaves, their antioxidant capacity, and their antibacterial and antifungal activities. Volatile compounds were identified using gas chromatography-mass spectrometry (GC-MS) coupled to solid-phase microextraction (SPME), while non-volatile compounds were annotated using UHPLC-MS/MS-ESI-Q-TOF. Antioxidant capacity was evaluated by DPPH assay, and antimicrobial activity was assessed in vitro against *Staphylococcus aureus*, *Escherichia coli*, *Pseudomonas aeruginosa*, and *Candida* species (*C. albicans*, *C. tropicalis*, *C. glabrata*). GC-MS analysis revealed 25 predominant volatile compounds, including ethyl dodecanoate, ethyl tetradecanoate, nonanoic acid, and 5-methylhexan-2-one, with documented antifungal, antioxidant, and antimicrobial activities. The crude extract and ethyl acetate fraction showed strong antioxidant capacity (EC_50_ 9.83 µg/mL and 6.42 µg/mL, respectively), and antifungal effects were observed against *Candida* species. This study provides the first comprehensive investigation of the antioxidant capacity and antibacterial and antifungal activities of *D. pubipetala*, together with a detailed chemical profile of its volatile compounds.

## 1. Introduction

The extraction of bioactive compounds from plant species is one of the oldest and most established applications of biodiversity [1,2]. The richness of Brazilian flora and fauna, coupled with the severe as a driving force in the search for new bioactive substances with diverse applications, such as compounds in cosmetics, flavors, fragrances, dyes and pigments, general food additives, components in insecticides and pesticides [3], or as important ingredients in various formulations for the treatment of infectious diseases [4].

Natural products derived from plants have served as medicine, given the broad efficiency of bioactive compounds against infectious agents [1,5]. The use of therapeutic agents based on plant derivatives is justified by the advancement of scientific knowledge in this area [6]. The lack of accessibility, the inefficacy of available drugs, and the high cost of antibiotics, especially as the occurrence of multidrug-resistant bacteria becomes increasingly common [7,8], are significant challenges.

A large part of the therapeutic action of plants is related to the bioactive properties of secondary compounds such as polyphenols, flavonoids, phenolic acids, terpenoids, saponins, alkaloids and others [3,5,7,9].

The Malpighiaceae, which is part of the Cerrado biome, one of the largest in Brazil [10], is composed of 45 genera, includes the genus *Banisteriopsis*, considered a sister genus to the species of interest in this investigation: *Diplopterys pubipetala* (A. Juss.) W. R. Anderson & C. Davis, commonly known as “marvaquero” (in northern Minas Gerais, Brazil), “cipó preto” [11], or “tucunacá” [12].

Regarding the species *D. pubipetala*, there are documented findings related to the botanical, anatomical, and physiological aspects of this liana [13,14,15,16,17]. Other areas of interest have gained significance, including the diversity of secondary metabolites revealed through phytochemical and histochemical tests, as well as the quantification of phenolic compounds and flavonoids, which showed values comparable to or even better than those of biologically significant substances previously quantified in the family [18,19].

In an *in vitro* analysis of anticancer activity, the ethyl acetate fraction from leaves and stems reduced the viability of B16F10 cells by over 50% at a concentration of 125 µg.mL^−1^, whereas other species widely used as chemotherapeutic agents failed to achieve such reduction in different cancer cell lines at this concentration [20]. Additionally, in tests of anti-inflammatory potential using the dichloromethane and ethyl acetate fractions on 3T3 mouse fibroblast cells exposed to ethyl acetate fractions, there was a decrease in the production of pro-inflammatory cytokines (TNF-α and IL-6) and an increase in the production of interleukin-10 and nitric oxide levels. These findings indicate the anti-inflammatory and immunoregulatory actions that may be exerted by the explored fraction [21].

In a study conducted on seeds, chromatographic analysis confirmed the presence of unsaturated fatty acids such as oleic, linoleic, and eicosanoic acids, which, similar to other species in the family, are already exploited by the industry. Furthermore, the species was reported as having promising potential in the oilseed sector due to the high content of these substances extracted from the seeds [22].

*Banisteriopsis* and other genera belonging to the Cerrado biome, with more advanced studies based on biomonitoring [23,24,25,26], stand out through chromatographic and spectroscopic analyses.

Through ultra-high-performance liquid chromatography coupled with mass spectrometry (UHPLC-MS), secondary metabolites have been identified in stem and leaf extracts of *D. pubipetala*, with the presence of alkaloids, flavonoids, terpenoids, saponins, and lactones documented in the literature [18,19]. However, there are still gaps, both in the study of non-volatile substances and in the investigation of possible volatile substances present in this species.

In this context, gas chromatography (GC) emerges as an essential technique due to its high precision and efficiency in identifying and quantifying classes of compounds such as terpenes, ketones, fatty acids, and other volatiles [27]. Knowledge of alternative methods related to the extraction of volatile components is essential, as each injection can vary depending on the type of sample analyzed. Therefore, liquid injection and solid-phase microextraction (SPME), with their specific characteristics, are widely used in volatile analyses [27,28].

The aim of this work is the characterization and evaluation of biologically active compounds from extracts and fractions of *D. pubipetala* leaves, focusing on antioxidant capacity, antibacterial and antifungal activities, as well as the annotation of substances in the EtOAc fraction by LC/MS and analyses using gas chromatography coupled to liquid injection mass spectrometry and SPME, including the analysis of fresh leaves. By characterizing its bioactive volatile compounds, this research highlights its relevant antifungal activity, contributing with valuable knowledge about future pharmacological applications of the species.

## 2. Results

### 2.1. Thin-Layer Chromatography (TLC)

The presence of flavonoids was verified by observing bands in a dark chamber under UV light (254 at 366 nm), with the predominant color being yellow, the retention factors were calculated: 026; 0.35; 0.49 and 0.61 (Figure S1). No co-migration occurred between the components of the crude extract and the ethyl acetate and methanol fractions in relation to the reference substance. Chromatographic tests conducted to detect alkaloids were not positive.

### 2.2. Evaluation of Antioxidant Capacity

The antioxidant capacity value for the hydroethanolic crude extract of *D. pubipetala* leaves was EC_50_ 9.83 ± 0.46 μg.mL⁻¹, indicating a high antioxidant capacity. In contrast, the effective concentration (EC_50_) obtained for the gallic acid standard was 2.10 ± 0.10 μg.mL⁻¹. Among the fractions, the lowest EC_50_ values were 6.46 ± 0.60 μg.mL⁻¹ for the ethyl acetate fraction (EtOAc) and 19.00 ± 3.58 μg.mL⁻¹ for the methanol fraction (MeOH). The fractions with the highest EC_50_ values, and thus the lowest antioxidant capacity, were the dichloromethane fraction (DCM) (52.90 ± 5.52 μg.mL⁻¹) and the n-hexane fraction (Hex) (84.50 ± 2.01 μg.mL⁻¹) (Table 1).

### 2.3. Antibacterial Activity

In this investigation, the best result for the biological inhibition assay of *S. aureus* was obtained with the ethyl acetate fraction minimum innibitory concentration (MIC 1000 µg.mL^−1^), which is considered weak. The other fractions, including the crude extract, exhibited MIC values equal to or greater than 2000 µg.mL^−1^.

The MIC values for all samples tested against *E. coli* and *P. aeruginosa* were equal to or greater than 2000 µg.mL^−1^, classifying them as inactive (Table 2).

### 2.4. Antifungal Activity

The results obtained for the extract and fractions of *D. pubipetala* showed fungistatic effects across all antifungal tests, with MIC < MFC, indicating that the minimum inhibitory concentrations were relatively low. This implies a very strong antimicrobial activity (<100 µg.mL^−1^) according to [30]. The highlights include the CE with a MIC of 3.9 µg.mL^−1^ against *C. albicans* and *C. tropicalis*, and the MeOH fraction with a MIC of 3.9 µg.mL^−1^ against *C. glabrata*.

Regarding the MIC/MFC ratio, the best result was observed for the EtOAc fraction, with MIC/MFC values of 31.25/500 µg.mL^−1^ for *C. albicans*, 7.81/500 µg.mL^−1^ for *C. glabrata*, and 15.6/1000 µg.mL^−1^ for *C. tropicalis*, followed by a promising result for the CE with a MIC/MFC ratio of 7.8/1000 µg.mL^−1^. These findings highlight a significantly superior antifungal activity compared to the antibacterial activity (Table 3).

### 2.5. LC-ESI-QTOF-MS/MS Analysis

In this research, substances from the EtOAc fraction were processed and identified based on exact masses and standard fragmentations using LC-ESI-QTOF-MS/MS in positive mode (Figure S2), following the previously described methodology. A total of 13 substances, including flavonoids and proanthocyanidins, were noted and are presented in Table 4.

### 2.6. GC/MS Analysis

The chromatographic analysis via liquid injection showed no significant differences among the samples. Some compounds detected in the less polar fractions (such as the hexane partition) were also present, albeit in smaller quantities, in the crude extract and the other fractions, with variations in their proportions. Therefore, the results presented in Table 5 pertain to the SPME analysis of the crude extract, fractions, and fresh leaf samples (Figures S3 and S7). The 25 substances detected correspond to the major peaks and include alcohols, aldehydes, ketones, acids, fatty acid esters, terpene derivatives, among others.

## 3. Discussion

### 3.1. Thin-Layer Chromatography (TLC)

Initial characterization of secondary metabolite groups allowed for the estimation of the relevance of a species producing compounds that could potentially become bioactive candidates and drive further investigations [5,31].

With chemical revelation using UV light and natural product reagent/poly ethylene glycol (NP/PEG) spray, the bands became even more evident at a wavelength of 254 nm due to the fact that all flavonoids cause fluorescence. The different colors at 365 nm (yellow, green, or blue) were attributed to the different structures of the compounds, as stated by [32,33]. In the ethyl acetate partition, the four distinct yellow fluorescent bands suggest the presence of flavonol glycosides. The retention factor (Rf) value of 0.26 also matches bands observed in the crude and methanolic extracts, indicating the possible presence of glycosylated quercetin and/or kaempferol. The Rf value of 0.35 suggests the presence of the flavonol glycoside rutin. Orientin and vitexin could be the glycosylated flavones indicated by the Rf value of 0.49 in the ethyl acetate partition sample. An Rf value of 0.61 was observed for a blue fluorescent band, which remains uninterpreted. In the crude extract, a band with an Rf value of 0.31 was detected, which may correspond to quercetin or kaempferol. All suggested identifications of compounds in this chromatographic analysis were based on Wagner and Bladt [33].

Phytochemical analyses with the hydroethanolic extract of *D. pubipetala* leaves revealed the presence of relevant metabolites such as phenolic compounds [18]. Glycosylated flavonoids had already been reported in another study with *D. pubipetala* [19], as well as for the species *Banisteriopsis argyrophylla* (Malpighiaceae) [34].

Regarding alkaloids, the use of mobile phase 1(MP1) and mobile phase 2(MP2) was aimed at detecting alkaloids derived from amino acids, as explored in the literature, particularly *β*-carbonyls present in extracts [35] from *Banisteriopsis caapi*, and also the possibility of purine alkaloids or pseudo-alkaloids. The absence of alkaloids was indicated by the lack of color change in the plates, consistent with other Malpighiaceae species that do not contain alkaloid compounds in their structures [36], such as *Heteropterys tomentosa*, and nitrogenous compounds as seen in the phytochemical prospecting of hydroethanolic leaf extracts and fractions of various polarities from *Banisteriopsis laevifolia* [26]. The variability in the presence of alkaloids in Malpighiaceae is recurring; a study found that both young and older stems of *B. caapi* exhibited a weak reaction for alkaloid detection, even though this class of phytochemicals is more common in *Banisteriopsis*, *Diplopterys*, *Tetrapterys*, and *Stigmaphyllon* [12].

The species *D. pubipetala* is used analogously to *B. caapi* in the preparation of Ayahuasca tea, particularly due to its production of alkaloids [37]. It is involved in traditional indigenous rituals in the Western Amazon region, providing experienced individuals with sensations recognized as mystical and spiritual experiences due to the presence of *β*-carbonyl alkaloids: harmine, harmaline, tetrahydroharmine, and harmol [35], along with increased serotonin concentrations and availability of N,N-dimethyltryptamine (DMT) facilitated by mixing with another species [12]. Investigations treat the components of this tea as responsible for aiding in recovery from Parkinson’s disease and Alzheimer’s disease [38].

Psychotherapeutic results of this tea support improvements in behavioral health, emotional regulation, and cognitive flexibility [39]. 

### 3.2. Evaluation of Antioxidant Capacity

In the analysis of variance applied to the samples, the EC_50_ values obtained for each measurement of antioxidant capacity in each partition met the assumptions (with *p* = 0.05). At a 5% significance level, the analysis indicated significant differences in the EC_50_ behavior among the different partitions used in the study. The Tukey test was used for multiple comparisons.

The values for the crude extract and the ethyl acetate and n-butanol (the most polar) fractions obtained from the leaf extract of *B. laevifolia* [24] and *B. argyrophylla* [25] were compatible, with EC_50_ values of 4.5 ± 0.9 and 4.3 ± 0.8 μg.mL⁻¹ for the crude extract, respectively. Similarly, the previously mentioned fractions also had the lowest EC_50_ values, being 4.1 ± 0.5 and 8.4 ± 1.0 μg.mL⁻¹ for *B. laevifolia* and 4.1 ± 0.1 and 4.8 ± 0.1 μg.mL⁻¹ for *B. argyrophylla*. For *Banisteriopsis oxyclada*, the value of the crude extract was 25.90 ± 1.10 μg.mL⁻¹, and the best relative values for the fractions corresponded to the ethyl acetate fraction (EC_50_ = 6.50 ± 0.40 μg.mL⁻¹) and the n-butanol fraction (EC_50_ = 6.80 ± 0.80 μg.mL⁻¹) [29].

The calculated antioxidant activity index (AAI) values for the gallic acid standard (19.05), the CE (4.07), and the EtOAc (6.23) and MeOH (2.13) fractions were the best, indicating a very strong antioxidant activity they are greater than 2.0 [40,41]. The AAI values for the DCM (0.76) and Hex (0.48) fractions suggested moderate to poor antioxidant activity.

The AAI relates the 2,2-diphenyl-1-picrylhydrazyl (DPPH) concentration used in the experiment with the EC_50_ of the sample, the result is a constant for each analyzed material that if obtained under the same conditions, that is, same extraction procedures or collection sites, should yield similar or very close results [41]. In this study, it was observed that the fractions with the highest AAI values were the most polar ones, in which the most active and major compounds are phenolics, such as flavonoids. This highlights their ability to donate hydrogen to a free radical to mitigate potential damage [26]. The EC_50_ results, similar to the investigations of extracts and fractions of the leaves of the aforementioned *Banisteriopsis*, suggest that the best EC_50_ values are related to the clear discrepancies in the polarities of the fractions.

### 3.3. Antibacterial Activity

Extracts exhibiting MIC values below 100 µg.mL^−1^ are considered to have very strong antimicrobial activity; when MIC values are between 100 and 500 µg.mL^−1^, the antimicrobial activity is moderate; from 500 to 1000 µg.mL^−1^, the antimicrobial activity is weak; and above 1000 µg.mL^−1^, the extract is considered inactive [30,42].

Studies on volatile essential oils from *B. campestris* flowers and leaf extracts of *B. laevifolia* and *B. oxyclada* have demonstrated moderate to very strong antimicrobial activity against *S. aureus*, particularly for oral diseases like caries and periodontitis [43]. The ethanolic leaf extract of *B. anisandra* showed significant inhibition against *S. aureus* [23,44].

In contrast, investigations into inhibition activity against *S. aureus* and *P. aeruginosa* did not result in microbial growth inhibition for *B. anisandra* extracts [45]. Research on extracts from *Byrsonima crassifolia* fruit parts found that the greatest antimicrobial potential was observed against gram-positive bacteria (*S. aureus*), particularly in ethanolic extracts (pulp and peel), while ether extracts did not inhibit the same bacterial growth [46]. Additionally, methanolic extracts from stems and roots of *Byrsonima* species also demonstrated significant inhibition against *S. aureus*, *E. coli*, and other bacteria [47].

Considering that *S. aureus* is a gram-positive bacterium, this observation can be related to the structural characteristics of the bacterial types involved, since *E. coli* and *P. aeruginosa* are gram-negative. It is well known that the structural characteristics of gram-negative and gram-positive bacteria provide differences in their antimicrobial activities in relation to plant extracts and other agents [48].

The absence of antibacterial activity, or the lower sensitivity of microorganisms, may also be due to the type of extract used and the quality of the plant material itself, which can be affected by seasonal conditions, soil, and bioactive content [48].

### 3.4. Antifungal Activity

To evaluate the MIC for *C. albicans*, *C. tropicalis*, and *C. glabrata*, a non-parametric data analysis was conducted, and the results indicated a significant difference (*p* = 0.00073) among the MIC across different samples.

This aligns with previous studies, such as the investigation on *Heteropterys aphrodisiaca* (Malpighiaceae) root extracts, which demonstrated stronger antifungal activity against *Candida* species with MIC values 125 to 250 µg.mL^−1^ (*C. albicans*, *C. parapsilosis*, *C. krusei*, *C. tropicalis*) than antibacterial activity against *S. aureus* where the minimal bactericidal concentration was between 250 and 500 µg.mL^−1^ [49].

Similarly, the aqueous leaf extracts of *B. anisandra* showed strong antifungal activity, particularly against *C. tropicalis*, with MIC values of 62.50 µg.mL^−1^ outperforming the standard antifungal agent fluconazole which exhibited a MIC > 64 µg.mL^−1^ [50]. Additionally, *C. albicans* displayed sensitivity to this plant’s extract in dilution tests [44].

Further studies involving the ethyl acetate fraction of *B. argyrophylla* indicated significant antifungal activity attributed to catechins, particularly against *C. glabrata*, with MIC values as low as 2.83 µg.mL^−1^. Other fractions containing quercetin and kaempferol demonstrated inhibitory activity against *C. albicans*, *C. glabrata*, and *C. tropicalis*, with MIC values ranging from 5.86 to 46.87 µg.mL^−1^ [25].

For the extracts and fractions of *B. laevifolia*, the ethanolic extract exhibited strong antifungal activity against *Candida* species, with MIC values ranging from 31 to 63 μg.mL^−1^. The n-butanol fraction showed moderate to strong activity (MIC ranging from 94 to 375 μg.mL^−1^), while the ethyl acetate fraction also exhibited strong activity (MIC ranging from 188 to 375 μg.mL^−1^) [24], likely due to the presence of metabolites such as flavonoids, phenolics, and tannins.

Promising results were also obtained using hydroalcoholic extracts of *Byrsonima crassifolia* leaves as a therapeutic alternative for fungal infections. Recently, a topical cream with antifungal properties against *C. parapsilosis* (MIC of 1 mg.mL^−1^) was developed [51].

The strong antifungal activity observed, particularly in the ethyl acetate fraction, may be attributed to the presence of flavonoids, phenolic compounds, and tannins, which are known to interfere with fungal cell wall synthesis, disrupt membrane integrity, and inhibit key enzymes involved in fungal metabolism [8,52]. This could suggest a mechanism of action similar to existing antifungal agents, such as azoles, which target ergosterol synthesis in fungal membranes [53]. However, compared to standard treatments like fluconazole, the observed MIC values indicate a potent antifungal effect, possibly offering an alternative with fewer side effects or resistance issues [49]. Further investigation into the specific interactions of these bioactive compounds with fungal targets could enhance understanding of their therapeutic potential and support the development of plant-based antifungal treatments [4,52].

### 3.5. LC-ESI-QTOF-MS/MS Analysis

Although liquid chromatography coupled with mass spectrometry is a powerful tool for the analysis and characterization of unknown compounds, reliable identification of chemical substances in plant material remains a challenge due to its complexity [54].

#### 3.5.1. Putative Identification of Flavonoids

Rutin is formed by the flavonoid quercetin aglycone along with the disaccharide rutinose at position 3 of the carbon ring [55]. C_27_H_30_O_16_ was identified from the protonated peak [M + H]^+^ with *m*/*z* 611.1612 and the two main fragments *m*/*z* 465.1032, corresponding to the loss of the rhamnoside [56], and *m*/*z* 303.0501, corresponding to quercetin aglycone [57].

Rutin is present in studies involving methanolic extract of *B. anisandra* and ethanolic extracts of roots and leaves of some *Heteropterys* species, and in extracts of ripe and unripe fruits of Malpighia species, among others [58]. This substance is associated with excellent antioxidant activity and pharmacological properties such as anti-inflammatory, hepatoprotective, antidiabetic, and neuroprotective effects [55].

The molecule isoquercitrin (quercetin-3-*O*-glucoside) C_21_H_20_O_12_, identified with the protonated peak [M + H]^+^ *m*/*z* 465.1032, had as main fragments *m*/*z* 303.0509 [M + H-Glu]^+^, *m*/*z* 153.0188 [M + H-Glu-H_2_O-C_8_H_6_O_2_]^+^ produced from Retro Diels-Alder (RDA) rearrangement, and *m*/*z* 137.0238 [M + H-Glu-O-H_2_O-C_8_H_6_O-O]^+^ in accordance with [59]. Isoquercitrin isolated from the ethyl acetate extract of *Byrsonima coccolobifolia* Kunth was evaluated in studies for its inhibition of NS2B-NS3 proteases essential in the Dengue virus (DENV) replication cycle, being considered a promising substance for developing strategies against this infection [60].

These two flavonoids (rutin and isoquercitrin) present in the *Byrsonima sericea* leaf extract were attributed with gastroprotective effects [61] and protection against ethanol-induced gastric lesions.

Isoorientin and orientin were also noted, with the formula C_21_H_20_O_11_ and the molecular ion [M + H]^+^ *m*/*z* 449.1084, showing losses of 120 *m*/*z* and 150 *m*/*z*, characteristic of mono-C-hexosyl portions in MS/MS, resulting in fragmented ions *m*/*z* 329.0667 and 299.0559, respectively, as described in the literature [62]. The presence of these substances has been reported in investigations related to Malpighiaceae, in extracts of *Bunchosia glandulifera* leaves and *Malpighia emarginata* DC fruits, associated with antioxidant effects [58].

The presence of vitexin and isovitexin (apigenin-C-hexoside) is related to the molecular ion [M + H]^+^ *m*/*z* 433.1141, corresponding to the formula C_21_H_20_O_10_, with observed losses of 120 *m*/*z* and 150 *m*/*z*; the resulting fragmented ions were *m*/*z* 313.0711 and *m*/*z* 283.0605, respectively, indicating the presence of these substances [62].

These substances orientin, isoorientin, vitexin, and isovitexin are present in extracts of *Bunchosia glandulifera* and *M. emarginata* DC leaves and are associated with antioxidant activity [58]. For *M. glabra* L., it was found that the extract, rich in such flavonoids, possesses properties that protect the liver against damage induced by toxic substances [63].

In this study, the presence of miricetin-3-*O*-galactoside or miricetin-3-*O*-glucoside was noted with *m*/*z* 481.0982 and formula C_21_H_20_O_13_. The fragments were 319.0447 *m*/*z*, suggesting cleavage at position 3 where glycosylation is characteristic, and 273.0399 *m*/*z* referring to the aglycone, both typical of 3-*O*-monoglycosides [6].

In some Malpighiaceae, such as *M. glabra* and *Byrsonima crassifolia* (L) Kunt., antioxidant, anti-inflammatory, and hepatoprotective effects related to substances like miricetin aglycone have been observed [58]. When comparing the antioxidant activity of miricetin-3-*O*-galactoside and miricetin aglycone [64], the EC_50_ is lower for miricetin aglycone, indicating greater oxidative power, implying that galactosylation negatively impacts the antioxidant process.

The putative identification of catechin and epicatechin was based on the ion [M + H]^+^ *m*/*z* 291.0864 and molecular formula C_15_H_14_O_6_. These compounds were considered isomers, corroborating with common fragmentations in the literature [65,66], such as *m*/*z* 165.0548, *m*/*z* 139.0390, and *m*/*z* 123.0440.

For the aerial parts extract of *B. caapi*, after decoction, considerable amounts of catechin and epicatechin were obtained, showing antimicrobial activity and action against pathogens [67]. Additionally, *B. caapi* extracts investigated for important chemical markers, including epicatechin and procyanidin B2, detected consistently in various parts of the plant across different seasons, were associated with high antioxidant value [68]. Epicatechin is also an inhibitor of the enzyme monoamine oxidase (MAO-B), and when combined with *ß*-carbolines, the potential synergistic effect may be valuable for neuroprotective activity.

The presence of catechins in leaves and stems of various *Byrsonima* species [58] has been associated with diverse activities including antileishmanial, antiulcer, antioxidant, antimicrobial, analgesic, anti-inflammatory, and others mentioned in studies.

Recently, among the flavonoids identified in *M. glabra* fruits, it was found that the quantification of epicatechin significantly increases in ripe fruits compared to green fruits. This increase is directly related to free radical scavenging activity [69].

Gallocatechin or epigallocatechin was also noted in this study, with the positive ion [M + H]^+^ *m*/*z* 307.0809. The characteristic fragmentations observed in this study align with the literature [65], with major cleavages resulting in *m*/*z* 181.0500 and *m*/*z* 139.0390. Flavonoids, including gallocatechin, investigated in *M. emarginata* leaves, stand out for their antioxidant and antifungal activities [58].

Another identified flavonoid has the positive ion [M + H]^+^ *m*/*z* 585.1257 and formula C_28_H_24_O_14_, with characteristic fragments of vitexin *O*-gallate. These fragments include: *m*/*z* 415.1028, *m*/*z* 313.0708, and *m*/*z* 153.0183, possibly resulting from the loss of the galloyl group, cleavage of the cross-ring hexose portion, and the galloyl fragment, respectively, as reported in the literature [70].

#### 3.5.2. Putative Identification of Procyanidins

Condensed tannins, known as proanthocyanidins, were noted in this study, specifically procyanidins (PAs), as the monomer units are solely (epi)catechins [71].

The putative identification of procyanidin B was carried out, with the observation of procyanidin dimers, where the prominent molecular cation was [M + H]^+^ at *m*/*z* 579.1502. The cleavage of interflavonoid bonds quinone methide (QM) allowed for the formation of the catechin monomer at *m*/*z* 289. Additional fragmentations were observed, producing the cation at *m*/*z* 427.1024, indicating the RDA cleavage of the flavonoid core (considered a standard fragmentation pattern for procyanidin B dimers), with subsequent loss of water (from ring C), resulting in the ion at *m*/*z* 409.0915 [71,72].

In this study, the *m*/*z* values of fragments obtained from positive ion electrospray ionization MS/MS were consistent with those reported in the literature. The most significant fragments were formed from the RDA reaction, QM cleavage, heterocyclic ring fission (HRF) and benzofuran formation (BFF) [71,73].

Similar to the literature [74], other *m*/*z* values of precursor ions for PA oligomers were noted, including *m*/*z* 867.2131 compatible with PA trimers, *m*/*z* 1157.2922 and *m*/*z* 1155.2775 compatible with PA tetramers, and *m*/*z* 1307.2887 compatible with gallic-acid-substituted PA tetramers. Additionally, pseudomolecular ions, common for large molecule fragmentations with low intensities, were observed throughout the analysis.

It is known that the mixed stereochemistry of PAs and their various degrees of polymerization promote different biological activities [72]. Among Malpighiaceae, PAs have been reported in *B. argyrophylla*, *B. caapi*, *Byrsonima verbascifolia* (L.) DC., *M. glabra* L. (Syn. *Malpighia punicifolia* L.), and *M. emarginata* DC. [58], generally associated with antioxidant potential, including free radical scavenging and subsequent preventive effects against possible diseases, including neurodegenerative conditions, as well as antibacterial, antiviral, and anti-inflammatory effects [58,71].

### 3.6. GC/MS Analysis

The methanolic partition analysis by GC was not performed, as this partition is predominantly composed of substances with higher polarity, such as flavonoids, phenolic acids, and other secondary metabolites, which have a high affinity for polar solvents and low volatility [75].

Understanding alternative methods related to the extraction of volatile components is essential, as each injection can vary depending on the type of sample analyzed. Thus, SPME was chosen, as it is suitable for the detection of volatile compounds, which tend to be non-polar or of low polarity. It is a simple and rapid method that uses a small amount of sample, and is applicable in various fields including environmental, food, aroma, pharmaceutical, forensic, and toxicological analysis [76]. The accurate identification of both similar and dissimilar compounds among the samples enabled the characterization of the volatile chemical profile of the leaves of *D. pubipetala*, as shown in Table 6.

Among the major compounds selected, the following can be highlighted.

Ethyl hexadecanoate was detected in all partitions and in the crude extract, with the highest concentration in the ethyl acetate partition. The biological activities associated with this metabolite include antimicrobial, antioxidant, nematicidal, hypocholesterolemic, and pesticidal actions, in addition to being used as a flavor enhancer, lubricant, and antiandrogenic agent [78]. It also acts in plants by preventing water loss during drought periods [79].

The presence of *S*-methyl-2-propenethioate was detected in fresh leaves and represented the largest area in the crude extract sample. The thiol-ester group allows for fatty acid synthesis and acyl group transfer, which are essential in pharmaceuticals as they can alter absorption, distribution, metabolism, excretion (ADME), and release of the active compound, thereby improving biological activity [77].

The ethyl esters, ethyl dodecanoate derived from lauric acid and ethyl tetradecanoate derived from myristic acid, as well as ethyl 3-hydroxy-2,2-dimethylbutanoate, possess antifungal potential, typical of natural fatty acids [80]. In the case of ethyl 3-hydroxy-2,2-dimethylbutanoate, its use as an excipient in pharmaceuticals has also been described in the literature to reduce unpleasant tastes, thereby improving flavor and palatability in medicine [81].

In general, several components detected and investigated also exhibit this biological activity: octanal [82], heneicosane [83], hex-3-en-2,5-diol [84], 5-methyl-hexan-2-one [85], all associated with antifungal activity. These compounds have potential applications in the agroindustrial and pharmaceutical sectors. Additionally, regarding potential applications in the agroindustrial sector, hexanoic acid is noteworthy for its inhibitory activity against fungi that spoil fruits [86], as well as the volatile 2,4-di-tert-butylphenol, which not only exhibits antifungal activity [87], but also has reported antioxidant [88] and larvicidal [89] activities. Nonanoic acid is commonly used as a herbicide and is also characterized by its antifungal action [90], which is recurrent in 2-butoxyethanol [91], dodecanal [92], and 6,10-dimethyl-5,9-undecadien-2-one [93].

Other detected substances exhibit potential for aromas and flavors, with applications in the food and pharmaceutical industries. These include 2,6,6-trimethylcyclohex-1-enecarboxaldehyde and nona-3,6-dien-1-ol, which are used in creating aromatic profiles [94]. Ethyl 3-methyl-cyclopentene-1-one, for example, enhances the flavor in sweeteners [95], while *α*-cis-3-hexenyl methylbutyrate is employed as a flavoring agent in pharmaceuticals [96].

The literature also points to the use of these substances in fragrance formulation for cosmetic products. 3-hexen-1-ol, found in high concentration in fresh leaves, is known for imparting a fresh aroma [97], and trans-*β*-ionone, which, in addition to its aroma, also exhibits antioxidant activity [98].

Additionally, 3-methyl-cyclopentano-1,2-dione shows limited inhibitory activity against the *Mycobacterium tuberculosis* H37Rv strain [99], while tetradecano exhibits antioxidant activity [100].

Two substances, 4,5-dimethyl-hept-2-en-3-ol and acetate of 10,13,13-trimethyl-tetradec-11-en-1-ol, have not yet been described for biological activities.

Throughout this investigation, it was observed that the substances detected exhibit significant antifungal activity. This finding reinforces the potential of *D. pubipetala* as a promising source of bioactive compounds, potentially useful in the treatment and prevention of fungal infections, as well as in the control of phytopathogens. This is particularly relevant given that the most commonly used control methods involve agrochemical products, which in turn cause environmental damage and pose risks to human and animal health [101]. In humans, the high resistance capacity of fungi complicates treatment methods, and some medications exhibit toxicity, as is the case with those used to treat candidiasis [102].

The use of GC-MS-SPME enabled a comprehensive characterization of the volatile chemical profile of the extract, partitions, and fresh leaves of *D. pubipetala*, which had not been previously investigated. Chromatographic analyses contributed to the distinction of substances across partitions, enabling the identification of both similar and dissimilar compounds among the samples. The major secondary metabolites identified are reported in the literature as possessing biological activities that, in addition to promoting intra- and intercellular interactions in organisms, contribute broadly to sectors such as agroindustry, pharmaceuticals, food, and cosmetics.

### 3.7. Statistical Analysis

#### 3.7.1. Antioxidant Capacity

The analysis of variance showed that there is a significant difference in absorbance behavior between at least two of the doses used. According to the Shapiro-Wilk test (at 5% significance), the residuals can be considered normal, and according to the Bartlett test (at 5% significance), they are homoscedastic. For this evaluation, the equations for the gallic acid standard, hydroethanolic extract, and the fractions of *D. pubipetala* leaves were used. The R^2^ values for all equations were greater than 0.98.

#### 3.7.2. EC_50_

The analysis of variance was applied to the EC_50_ values obtained from each repetition of the antioxidant capacity measurement for each sample. Since not all assumptions of the analysis of variance were met, a new approach was required. A “log” transformation was applied to the data, and a new analysis of variance was conducted. In this new analysis of variance, all assumptions were satisfied (with *p* = 0.05), and at a 5% significance level, the analysis indicated that there was a significant difference in the EC_50_ behavior among the different fractions used in this investigation. The Tukey test was used for multiple comparisons, with EC_50_ values being statistically different among all samples.

#### 3.7.3. Antimicrobial Activity

In the MIC assessment for bacterial species, the results of the triplicates involving all samples and microorganisms were identical, so there was no need to calculate the mean or standard deviation.

To evaluate the MIC for *Candida* species, a non-parametric analysis of the data was performed. Using *α* = 0.05, the Kruskal-Wallis test was applied. The results indicate a significant difference (*p* = 0.0073) between the MIC across the different fractions. In the subsequent multiple comparison test, Least Significant Difference (LSD), it was concluded that the sample with the lowest MIC was the crude extract for *C. albicans* and *C. tropicalis*, while for *C. glabrata*, the lowest MIC was found with the methanol fractions. In all cases, the results were significantly lower than those of the other samples.

## 4. Materials and Methods

### 4.1. Chemicals and Instruments

Reagents and chemicals used were analytical grade from Sigma Chemical Company (St. Louis, MO, USA).

For the experiments, the following equipment was used: a Nova Ética forced air circulation oven, model 400 - 4ND, Brazil; a Willey-type knife mill, model SL30, Solab, Brazil; a Shimadzu UV-VIS 2550 spectrophotometer for absorbance measurements.

For the UHPLC-MS/MS analysis, a Nexera UHPLC system from Shimadzu (Kyoto/Japan) coupled with a MaXis ETD high-resolution mass spectrometer—Bruker (Billerica, MA, USA) was used. For the Gas Chromatography-Mass Spectrometry (GC-MS) on a Shimadzu (Kyoto/Japan) GCMS-QP2020NX.

### 4.2. Plant Material

*Diplopterys pubipetala* leaves were collected in the Nova Esperança District, municipality of Montes Claros, Minas Gerais, Brazil, in December 2021 (16°36′07.0″ S 43°55′12.7″ W; https://maps.app.goo.gl/4NibPT6woJB29KK56 (accessed on 4 December 2021)). Herbarium specimens of the plant material were deposited in the herbarium Montes Claros under the number 4033. The study was registered in the National System for the Management of Genetic Heritage (SisGen) under the number A822A14.

### 4.3. Preparation of Plant Material

The leaves, after being washed with running water, were dried in a forced-air circulation oven at temperatures between 35 °C and 40 °C for seven days. Following this, the leaves were pulverized using a Willey-type knife mill. The resulting powder (800 g) was stored in paper bags under refrigeration (4 °C).

The hydroethanolic extract (7:3 *v/v*) was prepared using the exhaustive maceration method (250 g.L^−1^) and was stored in the dark at room temperature for seven days, with occasional stirring [103]. The mixture was then filtered, evaporated, and the resulting material was refrigerated (4 °C). The entire extraction process was repeated with the residue three more times, yielding 7.75% *w/w*.

Subsequently, liquid-liquid extraction was performed according to [104] was performed on a portion of the CE (18.85 g) in a hydroethanolic solution (7:3 *v/v*). Fractionation was carried out using two equal volumes (2 × 500 mL) of sequential solvents: n-hexane, dichloromethane, ethyl acetate, and methanol. Each fraction was dried in an oven at 38 °C with air circulation. The yield percentages for the respective fractions were 6.2% Hex, 13.0% for DCM, 15.3% for EtOAc, and 12.14% for MeOH. All samples were stored in a freezer until further use.

Healthy *D. pubipetala* leaves were stored in a Kraft paper bag and placed on ice, then kept in a freezer at −10 °C until chromatographic analysis.

### 4.4. Thin-Layer Chromatography (TLC)

Thin-layer chromatography (TLC) was performed on the CE, Hex, DCM, EtOAc, and MeOH fractions. The stationary phase (SP) consisted of aluminum plates pre-coated with silica gel 60, 0.2 mm thick, with a fluorescent indicator F_254_ (Macherey-Nagel/Düren-Germany). The aim was to detect compounds belonging to the flavonoid and alkaloid classes. The mobile phases were selected [33], with the mobile phase for flavonoids consisting of ethyl acetate: glacial acetic acid: formic acid: water (100:11:11:26) and two different mobile phases for alkaloids. The MP1 was composed of toluene: ethyl acetate: diethylamine (70:20:10); the MP2 was composed of ethyl acetate: methanol: water (100:13.5:10).

### 4.5. Evaluation of the Antioxidant Capacity of the Crude Extract and Fractions

The antioxidant capacity was assessed using the spectrophotometric method for radical scavenging by DPPH [105]. The concentrations of the crude extract and fractions (2.0 mL) varied between 2.0 and 95.0 µg.mL^−1^ and were added to 3.0 mL of a 40.0 µg.mL^−1^ ethanolic DPPH solution. The mixtures were stirred and kept in the dark for 30 min. Absorbance measurements were taken using a Shimadzu UV-Vis spectrophotometer (Kyoto/Japan) at 517 nm. Gallic acid (Neon) was used as a positive control at concentrations ranging from 0.8 to 2.4 µg.mL^−1^. Additionally, the absorbance of the ethanolic DPPH solution (negative control) was measured. The antioxidant capacity was calculated [106] using the linear equation and EC_50_ value (the concentration required to inhibit 50% of the DPPH radical). The AAI was calculated based on the ratio between the efficient concentration (EC_50_) and the concentration of the DPPH standard solution (40.0 µg.mL^−1^), when the value obtained for AAI is greater than 2, the result is considered strong [40]. The assays were performed in triplicate.

### 4.6. In Vitro Biological Assays for Antibacterial Activity

The antibacterial activity of the crude extract and fractions of *D. pubipetala* was evaluated against the microorganisms: *Staphylococcus aureus* (ATCC 6538), *Escherichia coli* (ATCC 8739), and *Pseudomonas aeruginosa* (ATCC 27853).

The MIC was determined using the broth microdilution method in 96-well microplates, performed in triplicate. The inoculum was adjusted for each bacterium to reach a cell concentration of 5 × 10^5^ colony-forming units per mL (CFU.mL^−1^), according to the Clinical and Laboratory Standards Institute [107].

Growth control (inoculated well) and sterility control (non-inoculated well free of antimicrobial agent) were included.

Tetracycline was used as a positive control (0.0115 to 5.9 μg.mL^−1^) and extract and fractions (0.98 μg.mL^−1^ to 2000 μg.mL^−1^). After sealing and incubation (24 h), 30 μL of a 0.02% aqueous resazurin solution was added to the microplates, which were then incubated for an additional 15 min at 37 °C. All tests were performed in triplicate.

### 4.7. In Vitro Biological Assays for Antifungal Activity

The antifungal activity of the crude extract and fractions of *D. pubipetala* was evaluated against the yeasts *Candida albicans* (ATCC 90028), *Candida tropicalis* (ATCC 13803) and *Candida glabrata* (ATCC 2001).

The assessment was conducted using the broth microdilution method according to the standards set by the Clinical and Laboratory Standards Institute [108].

Stock solutions of the samples were prepared in dimethyl sulfoxide (DMSO) (192.000 µg.mL^−1^). Working solutions were prepared by diluting the stock solutions in RPMI culture medium buffered to pH 7.2 with 3-N-morpholinopropanesulfonic acid (MOPS) (12.000 µg.mL^−1^). The inoculum was prepared using a spectrophotometric method and adjusted to match a McFarland scale of 0.5 to achieve a concentration of 6.0 × 10^6^ CFU.mL^−1^. Further dilutions in RPMI broth were made to reach a final inoculum concentration of 1.2 × 10^3^ CFU.mL^−1^.

The MIC was determined in 96-well microdilution plates, with serial dilutions of the samples ranging from 0.9 to 2000 µg.mL^−1^. RPMI broth with MOPS at a final pH of 7.2 was used as the culture medium. Each well was inoculated with 100 µL of the prepared inoculum, resulting in a final volume of 200 µL per well.

Amphotericin B (AMB) was used as the positive control, with concentrations ranging from 0.015 to 8.0 µg.mL^−1^. DMSO was used as the negative control at concentrations ranging from 5% to 3% (*v/v*) and extract and fractions (0.98 μg.mL^−1^ to 2000 μg.mL^−1^). A growth control was prepared with culture medium and inoculum without antifungal agents.

After incubation, 30 µL of a 0.02% aqueous resazurin solution was added to each well. The microplate was reincubated for an additional 3 h. The results were read visually, with blue and red colors indicating the absence and presence of microbial growth, respectively [109]. All tests were performed in triplicate.

### 4.8. Statistical Analysis

#### 4.8.1. Antioxidant Capacity

Analysis of variance (ANOVA) was conducted to assess the effects of different concentrations on absorbance results. Normality tests (Shapiro-Wilk) and tests for variance homogeneity (Bartlett’s test) were performed to verify the assumptions of the analysis. For cases where ANOVA indicates a significant difference between treatments, regression analysis will be conducted to study the relationship between variables. The analyses of variance were performed for the EC_50_ values obtained for each sample, with the Tukey test used for multiple comparisons.

#### 4.8.2. Antimicrobial Activity

For the evaluation of antimicrobial capacity, a non-parametric analysis of the data was performed. Using α = 0.05, the Kruskal-Wallis test was applied, followed by the LSD multiple comparison test.

### 4.9. Chemical Characterization by UHPLC-MS/MS

For the UHPLC-MS/MS analysis, the crude extract and fractions (5 mg) were prepared in safe-lock microtubes, solubilized in acetonitrile (HPLC grade). The samples were then placed in polypropylene vials at a final concentration of 5.0 mg.mL^−1^ (50 µL) in the automatic sampler rack for UHPLC-MS/MS analysis [110].

The UHPLC-MS/MS analysis was conducted using UHPLC system coupled with mass spectrometer equipped with electrospray ionization and quadrupole/time-of-flight (ESI-Q-TOF) detection, controlled by Compass 1.5 software (Bruker).

Aliquots of 2 µL were injected onto a Shimpack XR-ODSIII column (C18, 150 × 2.0 mm, 2.2 μm) (Shimadzu) at 40 °C with a flow rate of 400 μL.min^−1^. Mobile phases A and B (0.1% formic acid in water and acetonitrile, respectively) were used in a gradient elution, starting at 5% B for 5 min, followed by a linear ramp to 100% B over 45 min, and held at 100% B for 5 min. Mass spectra were acquired in positive ionization mode at a spectral rate of 5.0 Hz. Ion source parameters were set to 500 V end plate offset, 4500 V capillary voltage, 3.0 Bar nebulizer pressure, and drying gas flow and temperature of 8.0 L.min^−1^ and 200 °C, respectively. Fragmentation spectra were acquired in data-dependent mode (automated MS/MS) using a collision energy ramp between 15 and 60 eV. Ion cooler settings were optimized for a range of 100–1500 *m*/*z* using a sodium formate calibration solution (Sigma) at 1 mM in 50% 2-propanol (J. T. Baker, Phillipsburg, NJ, USA). Mass calibration was performed by initial infusion of the calibration solution (20 μL) into the ion source and recalibration post-acquisition of raw data. Compound detection was performed by analyzing chromatographic peaks, followed by formula determination based on exact mass and isotopic pattern (MS1). Putative identification was based on the comparison of compound fragment spectra (MS2) with reference spectra of an in-house database of standard compounds, the public spectra database MassBank [111], a list of targets specific to the plant’s family, genus and species as well as in silico fragment spectra generated from the Universal Natural Product Database—UNPD-ISDB [112]. Additionally, PubChem (https://pubchem.ncbi.nlm.nih.gov/) and Chemspider (http://www.chemspider.com/) databases were used to search for substance structures based on chemical formulas and fragment data, along with Sirius software 5.7.2.

### 4.10. Gas Chromatography-Mass Spectrometry

The analyses were conducted using Gas Chromatography-Mass Spectrometry (GC-MS) on a Shimadzu (Kyoto/Japan) GCMS-QP2020NX system equipped with an AOC 6000 auto-injector and a Restek SH-Rtx-5MS capillary column (30 m length, 0.25 mm ID, 0.25 µm film thickness). Liquid injection in hexane (1 µL) and SPME using a polydimethylsiloxane/divinylbenzene (PDMS/DVB) fiber (Supelco, Germany) were performed for volatile analysis on crude extracts, solvent partitions, and fresh leaf samples.

#### 4.10.1. Sample Preparation for Liquid Injection

Approximately 10 mg of dried samples from the hydroethanolic extraction phase were placed in a 12 mL vial, and 1 mL of HPLC-grade hexane (>96%) was added. The samples were dissolved, and 300 µL of the solution was transferred to the GC injection vial for analysis. Hexane served as a blank control.

#### 4.10.2. Sample Preparation for SPME Analysis

For fresh leaf analysis, chopped fragments were placed at the bottom of the vial. An empty vial from the same batch was used as the blank control.

#### 4.10.3. Experimental Conditions

For both liquid injection and SPME analyses, the column temperature program was set to an initial temperature of 40 °C, with a ramp of 10 °C/min up to 250 °C, which was held for one minute. The ion source temperature was set to 200 °C in the electron impact mass detector, with an interface temperature of 250 °C. Solvent cut-off occurred at 1 min, with data acquisition starting from 1 min and continuing to 22 min (total analysis time). The mass scan range was *m*/*z* 40 to 700 amu, with a scan speed of 3 scans/s. Helium gas (purity 5.0) was used at 100 kPa inlet pressure, a total flow of 4.8 mL/min, and the fiber was conditioned at 200 °C for 1 min prior to volatile adsorption [27].

## 5. Conclusions

The study highlighted the predominant antioxidant and antifungal activities of *Diplopterys pubipetala* and the identification of 25 volatile compounds through GC-MS, which may explain the observed antifungal action, as many of these compounds have been previously reported with such activity. The results reinforce the potential of this species for applications in pharmaceutical, agro-industrial, and cosmetic sectors. As a scientifically underexplored species, *D. pubipetala* presents promising opportunities for the isolation and characterization of individual bioactive compounds, as well as the evaluation of their mechanisms of action. Such efforts may lead to the development of new therapeutic agents and industrial applications, increasing the added value of this species in the context of health and biotechnology.

## Figures and Tables

**Table 1:** Comparison of the antioxidant capacity of *D. pubipetala* extracts and partitions with species from the genus *Banisteriopsis*.

	EC_50_ (µg.mL^−1^)					
	Crude Extract	Hex	DCM	EtOA_c_	BuOH * or MeOH	References
*D. pubipetala*	9.83 ± 0.46 ^d^	84.50 ± 2.01 ^a^	52.90 ± 5.52 ^b^	6.46 ± 0.60 ^e^	19.00 ± 3.58 ^c^	Authors
*B. laevifolia*	4.5 ± 0.9	58.1 ± 1.4	9.1 ± 0.8	4.1 ± 0.5	8.4 ± 1.0	[24]
*B. argyophylla*	4.3 ± 0.8	12. 0 ± 2.0	17.0 ± 0.4	4.1 ± 0.1	4.8 ± 0.1	[25]
*B. oxyclada*	25.9 ± 1.10	160.70 ± 3.40	100.40 ± 3.90	6.50 ± 0.40	6.80 ± 0.80	[29]

* The most polar partition is either in butanol (BuOH) or MeOH; EC_50_ for gallic acid (positive control) = 2.10 ± 0.10 µg.mL^−1^. ^a, b, c, d, e^ Different letters for each sample indicate a significant difference.

**Table 2:** Results of the in vitro biological assays against bacteria: *Staphylococcus aureus*, *Escherichia coli*, *Pseudomonas aeruginosa*.

Sample	MIC/MBC		
	Results em µg.mL^−1^		
	*Staphylococcus aureus* ATCC 6538	*Escherichia coli* ATCC 8739	*Pseudomonas aeruginosa* ATCC 27853
CE	2000/>2000	2000/2000	2000/>2000
Hex	2000/>2000	>2000/>2000	2000/>2000
DCM	>2000/>2000	>2000/>2000	2000/>2000
EtOAc	1000/2000	>2000/>2000	2000/2000
MeOH	2000/2000	2000/2000	2000/>2000

MIC (Minimum innibitory concentration); MBC (Minimum bactericidal concentration); *Staphylococcus aureus* (ATCC 29213)—Gentamicin control 0.7375 µg.mL^−1^; *Escherichia coli* (ATCC 25922)—Gentamicin control 1.0 µg.mL^−1^ (used as technique control).

**Table 3:** Contains the results of the in vitro biological assays against fungi: *Candida albicans*, *Candida tropicalis*, *Candida glabrata*.

Sample	MIC/MFC		
	Results em µg.mL^−1^		
	*Candida albicans* ATCC 90028	*Candida tropicalis* ATCC 13803	*Candida glabrata* ATCC 2001
CE	3.9 ^d^/2000	3.9 ^d^/2000	7.81 ^b^/500
Hex	31.25 ^b^/2000	62.5 ^a^/2000	15.6 ^a^/2000
DCM	15.6 ^c^/2000	31.25 ^b^/2000	7.81 ^b^/2000
EtOAc	31.25 ^b^/500	15.6 ^c^/1000	7.81 ^b^/500
MeOH	62.5 ^a^/2000	15.6 ^c^/2000	3.9 ^c^/2000

MIC (Minimum innibitory concentration); MFC (Minimum fungicidal concentration); ^a,b,c,d^ Different letters for each sample indicate a significant difference, as analyses were performed separately for each species; *Candida krusei* (ATCC 6258)—Amphotericin control 1.0 µg.mL^−1^; *Candida parapsilosis* (ATCC 22019)—Amphotericin control 0.5 µg.mL^−1.^ (used as technique control).

**Table 4:** Compounds annotated by UHPLC-ESI-MS/MS in ethyl acetate fraction from leaves of *Diplopterys pubipetala*.

Peak Identification	Compound Annotaded (Monoisotopic Mass)	RT (min)	MS [M + H]^+^ (*m*/*z*)	MS/MS Fragments (*m*/*z*)	Molecular Formula	Chemical Class
17	Epigallocatechin or Gallocatechin ^c^ (306.0740)	9.0	307.0809	307.0809; 181.0500; 163.0386; 139.0390	C_15_H_14_O_7_	Flavonoid
25	Procyanidin B1 ^a^ (578.1424)	11.4	579,1483	579,1502; 409.0927; 287.0567; 10402	C_30_H_26_O_12_	Proanthocyanidin
28	Epicatechin or Catechin ^b^ (290.0790)	11.7	291.0864	291.0864; 207.0655; 165.0548; 147.0441	C_15_H_14_O_6_	Flavonoid
30	Procyanidin Trimer ^c^ (866.2058)	12.2	867.2126	867.2126; 715.1665; 579.1498; 427.1024	C_45_H_38_O_18_	Proanthocyanidin
43	Orientin or Isoorientin ^a^ (448.1006)	13.9	449.1084	449.1084; 431.0990; 395.0775; 353.0667	C_21_H_20_O_11_	Flavonoid
44	Myricetin-3-O-galactoside ^a^ (480.0904)	14.1	481.0982	481.0982; 319.0447; 273.0399; 245.0450	C_21_H_20_O_13_	Flavonoid
50	Rutin ^b^ (610.1534)	14.7	611.1612	611.1612; 465.1032; 303.0501	C_27_H_30_O_16_	Flavonoid
51	Vitexin or Isovitexin ^b^ (432.1056)	14.9	433.1141	433.1141; 313.0711; 283.0605	C_21_H_20_O_10_	Flavonoid
52	Quercetin-3-O-Glucoside or Isoquercitrin ^b^ (464.0955)	15.1	465.1032	465.1032; 303.0509; 229.0499; 153.0188	C_21_H_20_O_12_	Flavonoid
58	Vitexin-O-gallate ^c^ (584.1166)	15.7	585.1257	585.1257; 415.1028; 313.0708	C_28_H_24_O_14_	Flavonoid

^a^ Mass-Bank, precursor ion [M + H]^+^ and product ion mass spectrum matched with those recorded at “Mass-Bank” MS/MS library; ^b^ In-house, precursor ion [M + H]^+^ and product ion mass spectrum matched with those recorded at “In-house” MS/MS library. ^c^ Target list, precursor ion [M + H]^+^ mass spectrum matched with those recorded on a list of targets specific to the plant’s family, genus, and species.

**Table 5:** Major Chromatographic peaks identified in the crude extract, fractions, and fresh leaves via SPME chromatographic analysis.

Substances	Area %	tR (min)
Crude Extract		
S-Methyl-2-propenethioate	35.02	8.126
Ethyl hexadecanoate	26.57	19.301
Ethyl-3-hydroxy-2,2-dimethylbutanoate	4.70	9.571
Ethyl dodecanoate	4.03	14.793
Ethyl tetradecanoate	2.10	17.155
Partition Hexane		
3-Methyl-2-cyclopenten-1-one	20.86	5.682
Ethyl hexadecanoate	18.92	19.308
Heneicosane	5.04	14.846
Tetradecane	4.47	12.226
3-Methyl-cyclopentane-1,2-dione	2.35	7.721
Partition Dichloromethane		
Hex-3-ene-2,5-diol	16.67	6.224
Ethyl hexadecanoate	13.83	19.311
5-Methylhexan-2-one	7.92	5.736
4,5-dimethylhept-2-en-3-ol	6.36	6.830
Nonanoic acid	4.66	10.464
Partition Ethyl Acetate		
Ethyl hexadecanoate	35.00	19.303
2-Butoxyethanol	19.63	4.720
2,4-Di-tert-butylphenol	8.19	13.818
10,13,13-trimethyl-tetradec-11-en-1-ol acetate	2.85	13.300
Nonanoic acid	1.52	10.326
Fresh Leave		
3-Hexen-1-ol	47.98	4.002
Ethyl 3-hydroxy-2,2-dimethylbutanoate	4.05	9.558
Dodecanal	3.39	12.386
Octanal	3.37	6.20
Nona-3,6-dien-1-ol	2.94	8.677
trans-β-ionone	2.62	13.556
2,6,6-trimethylcyclohex-1-enecarboxaldehyde	2.58	9.768
cis-3-hexenyl α-methylbutyrate	1.25	9.812
6,10-dimethyl-5,9-undecadien-2-one	1.17	13.005
Hexanoic acid	1.10	5.781
S-Methyl-2-propenothioate	1.00	8.083

**Table 6:** Major volatile substances detected by GC-MS-SPME and their functions highlighted in the literature.

Substance	Function	References
*S*-Methyl-2-propenethioate	Fatty acid synthesis Acyl group transfer Influence on ADME parameters Release of the active compound	[77]
Ethyl hexadecanoate	Antimicrobial Antioxidant Flavor enhancer Prevents water loss in plants	[78] [79]
Ethyl dodecanoate	Antifungal activity	[80]
Ethyl tetradecanoate	Antifungal activity	[80]
Ethyl-3-hydroxy-2,2-dimethylbutanoate	Antifungal activity	[80]
	Excipients in drugs	[81]
Octanal	Antifungal activity Potential use in agroindustry and pharmaceuticals	[82]
Heneicosane	Antifungal activity Potential use in agroindustry and pharmaceuticals	[83]
Hex-3-ene-2,5-diol	Antifungal activityPotential use in agroindustry and pharmaceuticals	[84]
5-Methyl-hexan-2-one	Antifungal activity Potential use in agroindustry and pharmaceuticals	[85]
Hexanoic acid	Antifungal activity	[86]
2,4-Di-tert-butylphenol	Antifungal activity	[87]
	Antioxidant	[88]
	Larvicidal	[89]
Nonanoic acid	Herbicidal Antifungal activity	[90]
2-Butoxyethanol	Antifungal action	[91]
Dodecanal	Antifungal action	[92]
6,10-dimethyl-5,9-undecadien-2-one	Antifungal action	[93]
2,6,6-trimethylcyclohex-1-enecarboxaldehyde	Aromas and flavors Application in the food and pharmaceutical industries	[94]
Nona-3,6-dien-1-ol	Aromas and flavors Application in the food and pharmaceutical industries	[94]
3-Methyl-2-cyclopenten-1-one	Flavor enhancer	[95]
cis-3-hexenyl α-methylbutyrate	Flavoring in pharmaceuticals	[96]
3-Hexen-1-ol	Cosmetic formulation	[97]
trans-*β*-Ionone	Aroma Antioxidant activity	[98]
3-Methyl-cyclopentane-1,2-dione	Mycobactericidal activity	[99]
Tetradecane	Antioxidant activity	[100]
4,5-dimethylhept-2-en-3-ol	No described biological activity	--
10,13,13-trimethyl-tetradec-11-en-1-ol acetate	No described biological activity	--

## Data Availability

All research data are already available in the article itself and in the supplementary material. However, additional information can be provided if necessary via email by the corresponding author.

## Supplementary Materials

The following supporting information can be downloaded at: https://www.mdpi.com/article/10.3390/molecules30040946/s1, Figure S1: Chromatoplate for detection of flavonoids; Figure S2: Chromatographic analysis LC-ESI-QTOF-MS/MS in the ethyl acetate partition of *D. pubipetala*.; Figure S3: Volatile compounds in the crude extract of *D. pubipetala* identified by GC-MS coupled to SPME.; Figure S4: Volatile compounds in the hexane partition of *D. pubipetala* identified by GC-MS coupled to SPME.; Figure S5: Volatile compounds in the dichloromethane partition of *D. pubipetala* identified by GC-MS coupled with SPME.; Figure S6: Volatile compounds in the ethyl acetate partition of *D. pubipetala* identified by GC-MS coupled with SPME.; Figure S7: Volatile compounds in fresh leaves of *D. pubipetala* identified by GC-MS coupled with SPME.
