# Supplementary material for: Diplopterys pubipetala (Malpighiaceae): Insights into Antioxidant, Antibacterial, and Antifungal Activities with Chemical Composition Analysis via UHPLC-MS/MS and GC/MS
Source: Molecules. 2025 Feb 18;30(4):946. doi: 10.3390/molecules30040946 (PMC11858539; doi:10.3390/molecules30040946)

Supplementary Materials

Figure S1: Chromatoplate for detection of flavonoids.

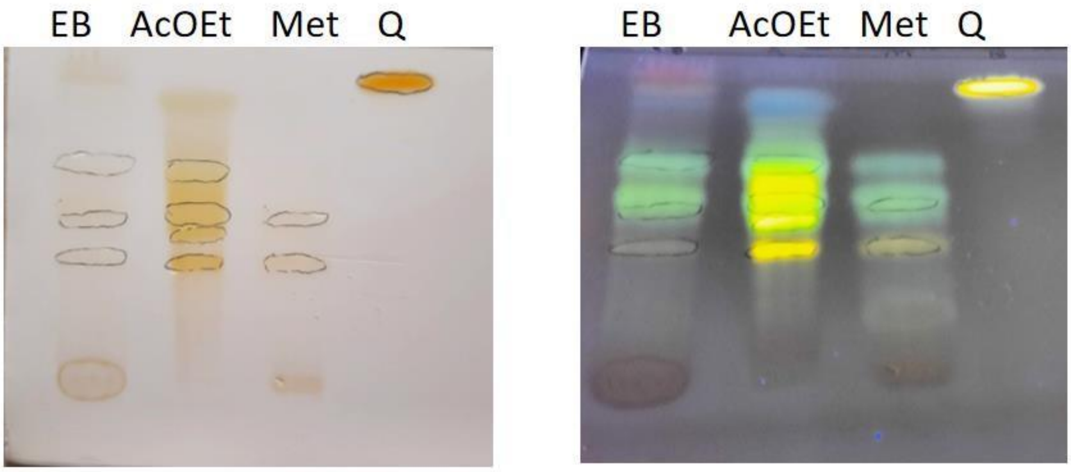

A) NP-PEG Developing agent. B) NP-PEG Developing agent (UV_{365})

Figure S2: UHPLC-ESI-QTOF-MS/MS profile of the ethyl acetate fraction from *Diplopterys pubipetala* leaves. Chromatograms registered in positive ionization mode (ESI⁺) showing all compounds detected from 0 to 60 min (A) and the substances annotaded from 8.6 to 16.2 min (B). Annotated compounds: see Table 4. Chromatographic and spectrometric conditions: see the materials and methods section.

(A)

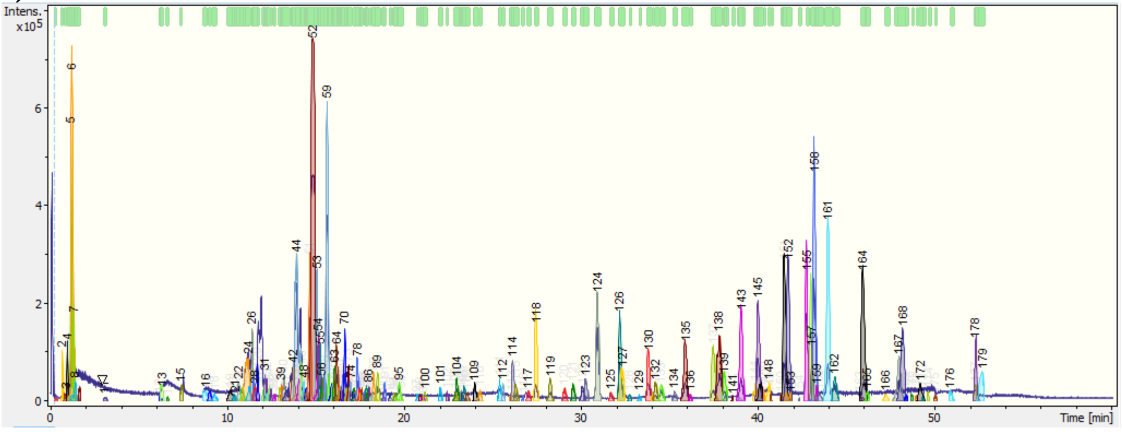

(B)

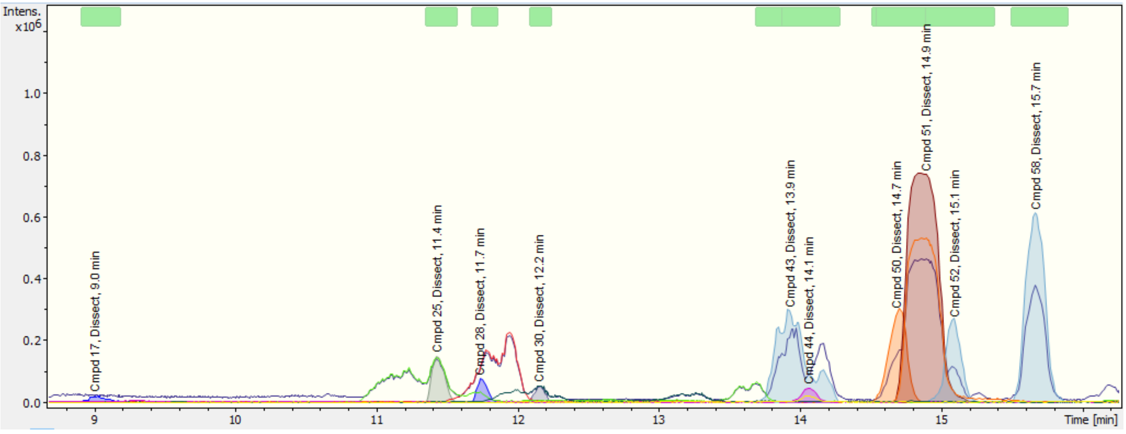

Intens.
x10^6
1.0
0.8
0.6
0.4
0.2
0.0
9
10
11
12
13
14
15
Time [min]
Cmpd 17, Dissect, 9.0 min
Cmpd 25, Dissect, 11.4 min
Cmpd 28, Dissect, 11.7 min
Cmpd 30, Dissect, 12.2 min
Cmpd 43, Dissect, 13.9 min
Cmpd 44, Dissect, 14.1 min
Cmpd 50, Dissect, 14.7 min
Cmpd 51, Dissect, 14.9 min
Cmpd 52, Dissect, 15.1 min
Cmpd 58, Dissect, 15.7 min

Figure S3: Volatile compounds in the crude extract of *D. pubipetala* identified by GC-MS coupled to SPME.

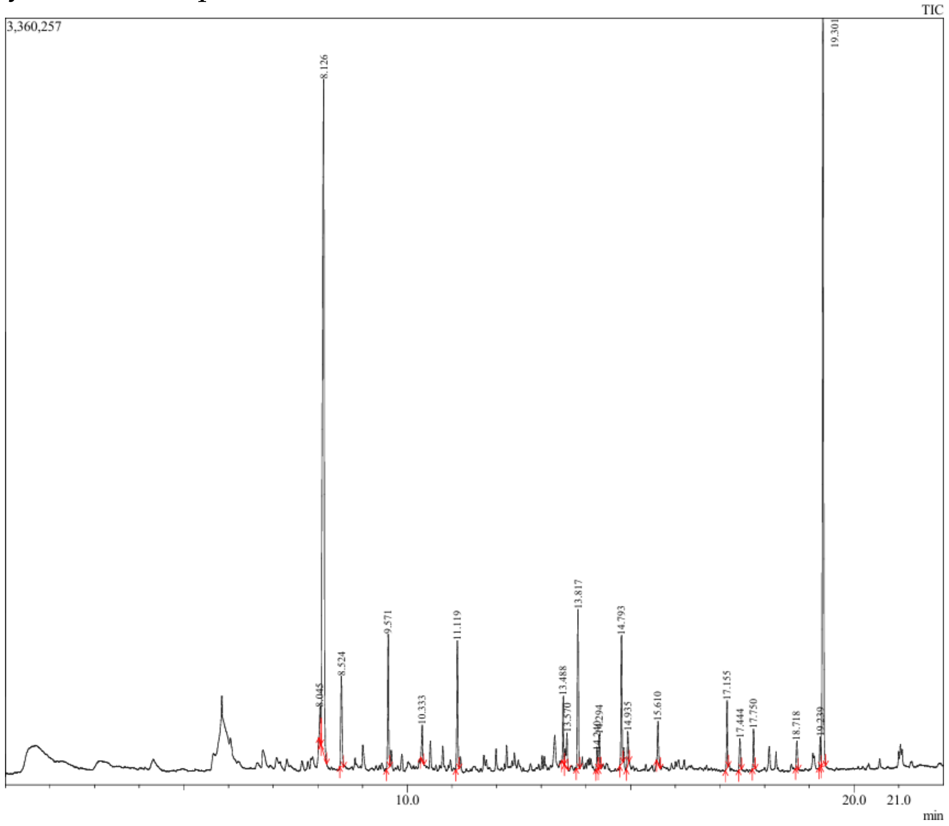

Figure S4: Volatile compounds in the hexane partition of *D. pubipetala* identified by GC-MS coupled to SPME.

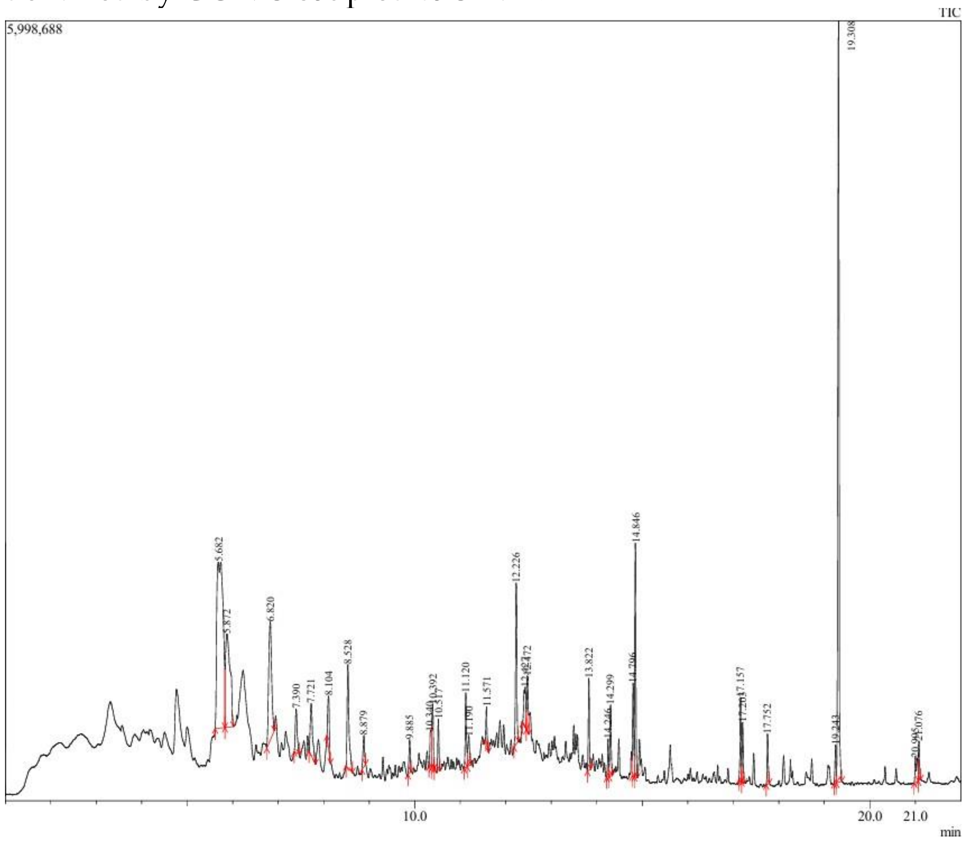

Figure S5: Volatile compounds in the dichloromethane partition of *D. pubipetala* identified by GC-MS coupled with SPME.

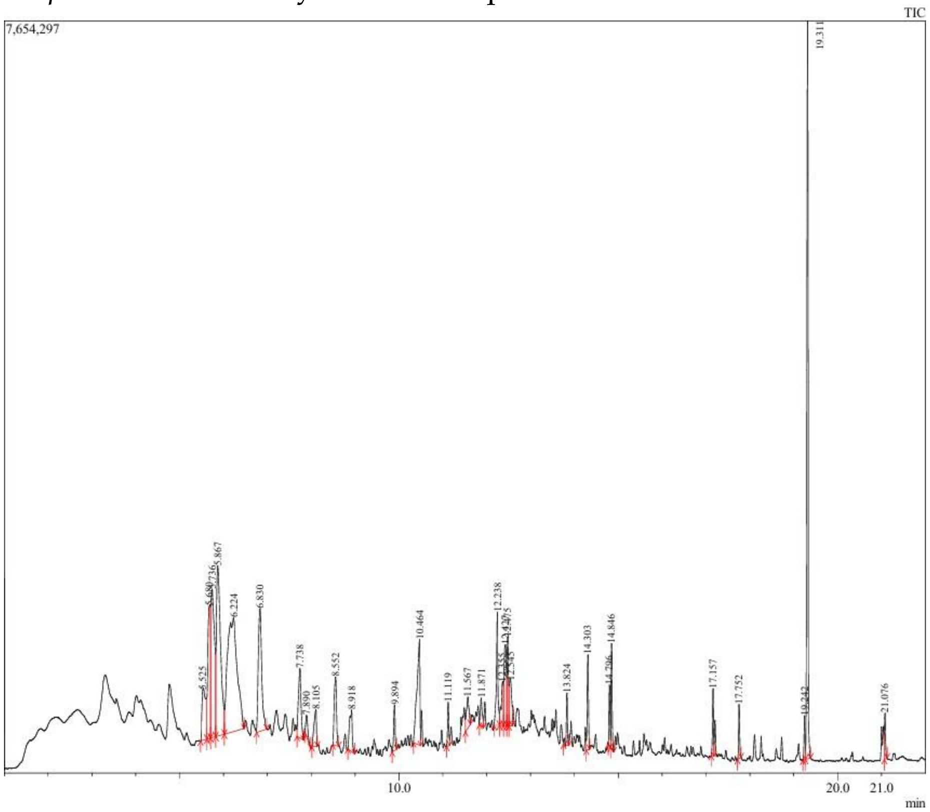

Figure S6: Volatile compounds in the ethyl acetate partition of *D. pubipetala* identified by GC-MS coupled with SPME.

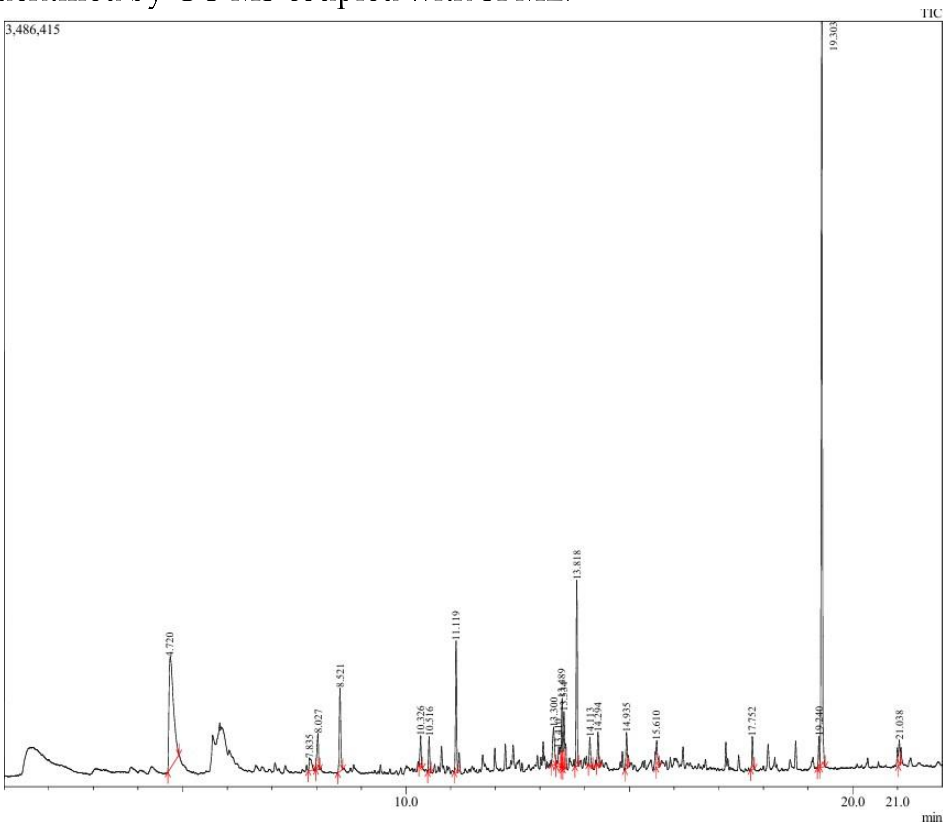

Figure S7: Volatile compounds in fresh leaves of *D. pubipetala* identified by GC-MS coupled with SPME.

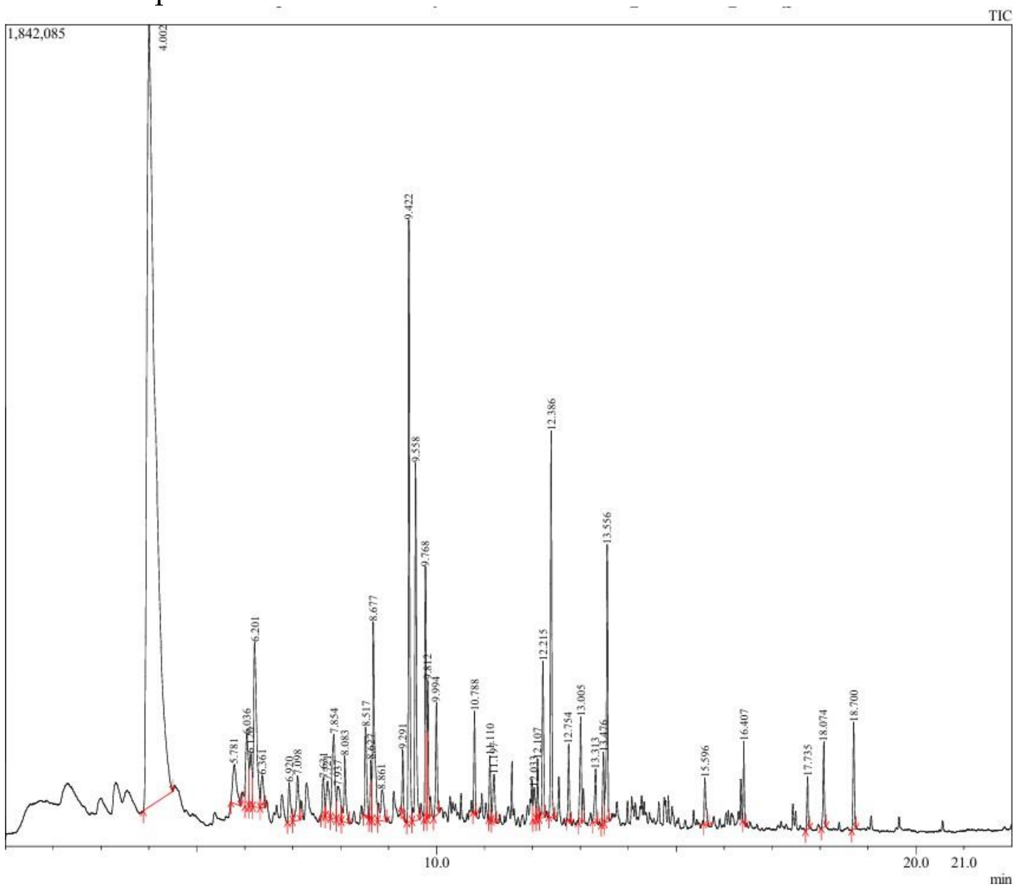

Supplement: Supplementary file 1 [file molecules-30-00946-s001.zip › molecules-3403415-supplementary.pdf]
